# Beneficial shift of rhizosphere soil nutrients and metabolites under a sugarcane/peanut intercropping system

**DOI:** 10.3389/fpls.2022.1018727

**Published:** 2022-12-02

**Authors:** Xiumei Tang, Yonglin He, Zheng Zhang, Haining Wu, Liangqiong He, Jing Jiang, Weiwei Meng, Zhipeng Huang, Faqian Xiong, Jing Liu, Ruichun Zhong, Zhuqiang Han, Shubo Wan, Ronghua Tang

**Affiliations:** ^1^ Cash Crops Research Institute, Guangxi Academy of Agricultural Sciences, Nanning, China; ^2^ Key Lab of Crop Genetic Improvement and Ecological Physiology of Shandong, Shandong Academy of Agricultural Sciences, Jinan, China

**Keywords:** peanut, sugarcane, intercropping, nutrient, metabolite

## Abstract

Intercropping systems have been studied as a sustainable agricultural planting pattern to increase soil quality and crop yields. However, the relationships between metabolites and soil physicochemical properties remain poorly understood under sugarcane/peanut intercropping system. Thus, we determined the rhizosphere soil physicochemical properties, and analyzed rhizosphere soil metabolites and root metabolites by metabolomics method under monoculture and intercropping patterns of sugarcane and peanut. The results showed that pH, the contents of total phosphorus (P), total potassium (K), available nitrogen (N), available phosphorus (P), and available potassium (K) were higher in rhizosphere soil of intercropping peanut than monoculture peanut, and the content of total P was higher in rhizosphere soil of intercropping sugarcane than monoculture sugarcane. Sugarcane/peanut intercropping also significantly increased the activities of acid phosphatase and urease in rhizosphere soil. The metabolomics results showed that 32 metabolites, mainly organic acids and their derivatives (25.00%), nucleotides and their metabolites (18.75%), were detected in root and rhizosphere soil samples. In the MP-S (rhizosphere soil of monoculture peanut) vs. IP-S (rhizosphere soil of intercropping peanut) comparison, 47 differential metabolites (42 upregulated) were screened, including glycerolipids (19.15%), organic acids and their derivatives (17.89%), and amino acids and their metabolites (12.77%). In the MS-S (rhizosphere soil of monoculture sugarcane) vs. IS-S (rhizosphere soil of intercropping sugarcane) comparison, 51 differential metabolites (26 upregulated) were screened, including heterocyclic compounds (15.69%), glycerolipids (11.76%), and organic acids and their derivatives (9.80%). The metabolite species from MP-S, MS-S, IP-S, and IS-S were similar, but some metabolite contents were significantly different, such as adenine, adenosine, maltotriose, thermozeaxanthin-13 and PE-NMe (20:0/24:0). Adenine and adenosine were detected in root and rhizosphere soils, and their levels were increased in the intercropping treatment, which were mainly related to enhanced purine metabolism in root and rhizosphere soils under the sugarcane/peanut intercropping system. Importantly, adenine and adenosine were significantly positively correlated with total P and total K contents, acid phosphatase and urease activities, and pH. This study clarified that the sugarcane/peanut intercropping system could improve soil nutrients and enzymes and was related to purine metabolism.

## 1 Introduction

Sugarcane (*Saccharum officinarum* L.) is the most important sugar crop and renewable energy crop and has been cultivated in more than 90 tropical and subtropical countries (Kusumawati et al., 2021). As a perennial plant, long-term sugarcane monoculture patterns seriously restrict replanting and yield, resulting in large economic losses (de Oliveira, 2018; Zhang and Govindaraju, 2018). Repeated planting of a single crop on the same field usually leads to serious continuous cropping obstacles, soil physicochemical property deterioration, allelopathy, and nutrient imbalance and finally decreases the yield and product quality of crops, all of which put agricultural sustainability at risk (Yang et al., 2015; Chen et al., 2020). Sugarcane/peanut intercropping can relieve continuous cropping obstacles for peanut and sugarcane, making full use of light, soil nutrients and land resources and increasing farmers’ economic benefits, which contributes to the development of efficient and sustainable production of sugarcane and peanut (Tang et al., 2021b).

Intercropping is widely used across the Americas, Asia, Africa, and Europe because it has been shown to improve crop growth and achieve high and stable yields (Li et al., 2018). However, not all intercropping patterns deliver yield benefits or other positive outcomes. For example, in a field experiment of maize/wheat intercropping, because wheat has greater nutrient (N, P, and K) acquisition ability, the root system of maize was restricted during the early stage, and the yield decreased significantly (Li et al., 2001). This suggests that intercropping crop species with equal nutrient utilization efficiencies may have a negative impact, such as cereal/cereal and legume/legume intercropping (He et al., 2013). Poaceae/legume crop intercropping has been widely studied in virtue of its niche complementarity and positive interspecific interactions in resource use (Li et al., 2014; Jiang et al., 2022) and has shown yield advantages, such as sugarcane/soybean (Luo et al., 2016), maize/peanut (Jiang et al., 2022), wheat/faba bean (Wahbi et al., 2016), and sugarcane/peanut (Tang et al., 2021a), etc. The facilitation role of N_2_ fixation played by legumes elevates the N supply level in the intercropping system and promotes the uptake of N by adjacent Poaceae crops (Li et al., 2009). Sugarcane is a kind of crop with wide row spacing and slow seedling growth and is suitable for intercropping with other crops that grow rapidly, such as peanut and soybean (Shen et al., 2018; Tang et al., 2021b). In particular, sugarcane/peanut intercropping significantly increased the economic benefit by 87.84% and 36.38% compared with that of monocultured peanut and monocultured sugarcane, respectively (Tang et al., 2021a). The facilitation mechanism of Poaceae/legume intercropping remains unclear, and some studies have showed that it may be related to soil nutrients, biological nitrogen fixation, microbial diversity, and exudates, etc.

Numerous studies have shown that intercropping significantly increases soil nutrients (e.g., total N, total P, available N, available P, available K, and organic matter) and soil enzyme activities (e.g., urease, protease, catalase, sucrose, acid phosphatase) (Luo et al., 2016; Li et al., 2018; Tang et al., 2021a; Tang et al., 2021b; Jiang et al., 2022). Intercropping can effectively improve the mobilization and uptake of nitrogen (N), phosphorus (P), potassium (K), and micronutrients *via* interspecific interactions in the rhizosphere (Inal et al., 2007; Zuo and Zhang, 2008; Li et al., 2014). In addition, legume/cereal intercropping systems can improve the utilization of phosphorus (P) by root exudation of organic acids from legume crops and improve legume nitrogen (N) uptake by enhanced nodulation of legume crops (Fang et al., 2006; Li et al., 2007). Several studies have revealed that the activity of soil enzymes and the effective nitrogen and phosphorus contents of soil are significantly increased in sugarcane/soybean intercropping (Li et al., 2012; Peng et al., 2014; Solanki et al., 2018).

The metabolites in soil are complex and widely sourced, including plant root exudates, microbial metabolites, and the decomposition of plants, microbes and soil organic matter (Cheng et al., 2018). Defining the composition of root exudates in soil remains a great challenge (White et al., 2017). Root exudates can improve the effective nutrient content because they can change the physicochemical conditions of the rhizospheric soil and activate soil nutrients by dissolving nutrients that are difficult to dissolve (Shi, 1993). In recent years, research on soil nutrients and root exudates has become a hot topic (Li et al., 2014). Although several attempts have been made to collect and analyze plant exudates using different methods, it is difficult to reflect the actual situation of soil in the field (White et al., 2017; Cheng et al., 2018). The distinction between soil and root metabolites still needs to be further explored. A new mechanism of overyielding was demonstrated in faba bean/maize intercropping, in which phosphorus mobilized by one crop species increases the growth of a second crop species grown in alternate rows, leading to large yield increases in phosphorus-deficient soils (Li et al., 2007). Faba bean mobilizes insoluble soil P by releasing protons, malate and citrate into the rhizosphere, thus promoting maize overyield (Li et al., 2007). Chickpea can also exude more acid phosphatase and decompose soil organic P into an inorganic form, which can be absorbed and utilized by the intercropping crop maize (Li et al., 2004). Peanuts secrete protons and organic acids to activate insoluble inorganic phosphorus, promoting the absorption of phosphorus in root rhizosphere soil, which is conclusively beneficial to the growth of both peanut and cassava (Lin et al., 2018; Liu et al., 2019).

Sugarcane/peanut intercropping, which can increase yield and economic income, is being popularized in southern China (Tang et al., 2021a; Tang et al., 2021b). To date, research on sugarcane/peanut intercropping mainly focuses on improving soil nutrients and rhizosphere microecology and increasing yield (Solanki et al., 2019; Tang et al., 2021a; Tang et al., 2021b; Pang et al., 2022). However, the changes in root metabolites in sugarcane/peanut intercropping and the relationship between metabolites and soil nutrients have not been studied. In this study, we determined and analyzed soil physicochemical properties, as well as metabolites, in peanut and sugarcane roots and their corresponding rhizosphere soils in monoculture and intercropping systems. The objectives of this paper are as follows: (i) investigate the changes in rhizosphere soil nutrients and enzyme activity under a sugarcane/peanut intercropping system, (ii) identify the differential metabolites of root and rhizosphere soils between monoculture and intercropping, and (iii) analyze the correlation between soil nutrients and metabolites under the sugarcane/peanut intercropping system. This study provides comparative metabolomic insights for evaluating the influence of sugarcane/peanut intercropping on the microecological environment.

## 2 Materials and methods

### 2.1 Experimental site and plant materials

The field site was previously planted with sugarcane. The experiments were performed from 2020 to 2021 at the Lijian Scientific Base (23°14′25″N, 108°03′42″E) of the Guangxi Academy of Agricultural Sciences, Nanning City, GZAR, China. The tested soil was sandy soil, with organic matter, total nitrogen (total N), total phosphorus (total P), total potassium (total K), available nitrogen (available N), available phosphorus (available P), and available potassium (available K) contents of 18.280 g/kg, 1.022 g/kg, 0.315 g/kg, 6.583 g/kg, 82.83 mg/kg, 120.78 mg/kg and 111.67 mg/kg, respectively. The pH was 7.02. The sugarcane variety “Guitang 42” and the shade-tolerant peanut variety “Guihua 836” were obtained from the Cash Crops Research Institute of the Guangxi Academy of Agricultural Sciences.

### 2.2 Experimental design

**Figure 1:**
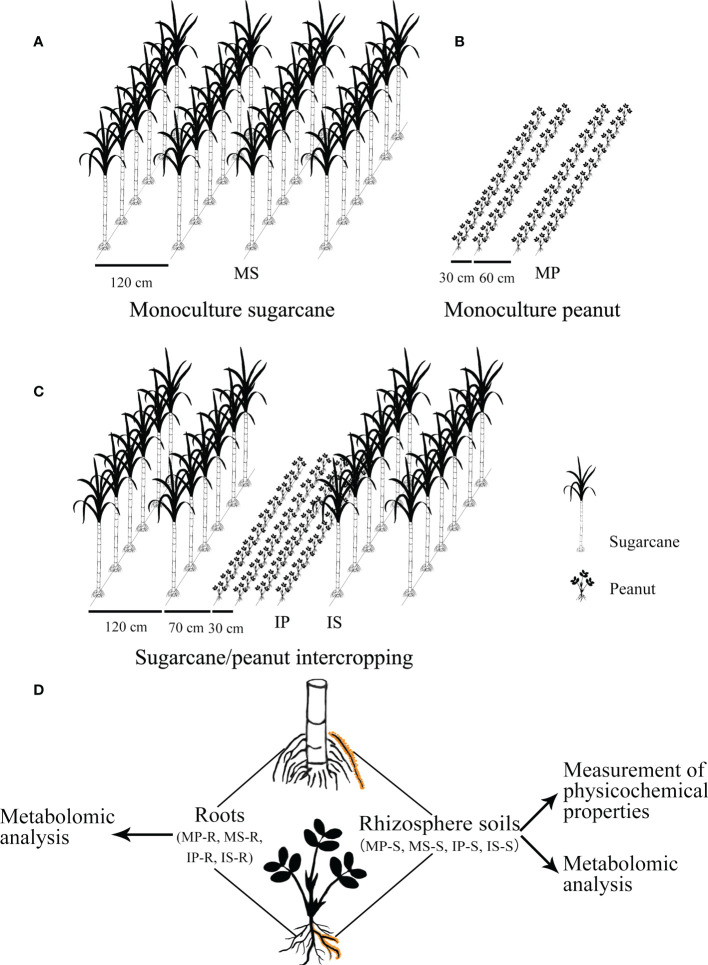
Experimental outline of the sugarcane and peanut field. **(A)** Monoculture sugarcane; **(B)** monoculture peanut; **(C)** sugarcane/peanut intercropping; **(D)** schematic figure of sampling and research route. MP-S, MS-S, IP-S and IS-S represent rhizosphere soils of monoculture peanut, monoculture sugarcane, intercropping peanut and intercropping sugarcane, respectively. MP-R, MS-R, IP-R and IS-R represent the roots of monoculture peanut, monoculture sugarcane, intercropping peanut and intercropping sugarcane, respectively.

The experiment was performed in plots (6 m × 8 m) in a randomized design with three replicates in each treatment. There were three planting treatments as follows: monoculture sugarcane (MS), monoculture peanut (MP), and sugarcane/peanut intercropping, including intercropping peanut (IP) and intercropping sugarcane (IS). The following method for crop layout was implemented (**Figure 1**): (i) Monoculture peanut adopted the pattern of wide–narrow rows, and the line spacing of wide rows and narrow rows was 0.6 m and 0.3 m, respectively; (ii) monoculture sugarcane was implemented with a line spacing of 1.2 m; (iii) sugarcane/peanut intercropping adopted the pattern of planting two lines of sugarcane and four lines of peanut, and the line spacing of wide rows and narrow rows was 1.2 m and 2.3 m in intercropping sugarcane, respectively. The line spacing between peanut and sugarcane was 0.7 m and the line spacing of intercropping peanut was 0.3 m.

### 2.3 Planting process and field management

On March 8th, 2020, sugarcane and peanut were planted simultaneously in the field. Fertilization management was carried out according to the absorption characteristics and actual amount of peanut and sugarcane because sugarcane requires much more fertilizer than peanut. All sugarcane treatments were uniformly applied in the planting furrow with 1500 kg/ha compound NPK granulated fertilizers (N-P_2_O_5_-K_2_O=15-15-15). All peanut treatments were uniformly applied in the planting furrow with 450 kg/ha compound NPK granulated fertilizers (N-P_2_O_5_-K_2_O=15-15-15) and 750 kg/ha fused calcium-magnesium phosphate fertilizer (available P_2_O_5_ 18%). Two irrigations were performed based on crop water requirements and soil water content. Pesticides and herbicides were applied approximately two months after sowing. On July 8th, 2020, peanut was harvested, and we hilled up soil around the roots of sugarcane and added 1500 kg/ha compound NPK granulated fertilizers to promote the rapid growth of sugarcane. When the sugarcane was sufficiently ripe, it was harvested on December 25th, 2020. We cut the stalks of the sugarcane, and their roots remained in the soil, which will grow into stubble cane the next year. On March 11th, 2021, peanut was still planted in the same position as the former year, which was intercropped with stubble cane. The areas of plots, fertilization and management were the same as those described for 2020. On July 12th, 2021, peanuts matured and were harvested, and we collected root and rhizosphere soil samples for analysis. The sugarcane continued to grow until harvest on December 27th, 2021.

### 2.4 Crop roots and rhizosphere soil collection

On July 12th, 2021, the time to harvest the mature peanuts. For each treatment, ten plants were randomly selected and dug out from each plot, the excess bulk soil was removed by gentle shaking, and the soil adhering to the root was considered rhizosphere soil. The rhizosphere soil of the sugarcane and peanut plants was collected with a sanitized soft brush, mixed and separated into two sealed virus-free bags, which were kept in an icebox and taken to the laboratory. One bag was dried naturally, ground and sieved to determine the nutrient content and soil enzyme activity. The other bag was maintained in the refrigerator at -80 °C and used for the extraction of rhizosphere soil metabolites, including rhizosphere soil of monoculture peanut (MP-S), rhizosphere soil of intercropping peanut (IP-S), rhizosphere soil of monoculture sugarcane (MS-S), and rhizosphere soil of intercropping sugarcane (IS-S). Roots were used for metabolite extraction, including roots of monoculture peanut (MP-R), roots of intercropping peanut (IP-R), roots of monoculture sugarcane (MS-R), and roots of monoculture sugarcane (IS-R). Rhizosphere soil metabolites are more complex and abundant than root metabolites. Therefore, we selected nontargeted metabolomics with high throughput and wide coverage for the analysis of rhizosphere soil metabolites and selected widely targeted metabolomics with high sensitivity, quasi-quantitative and quasi-qualitative for the analysis of root metabolites.

### 2.5 Measurement of rhizosphere soil physicochemical properties

According to previously reported methods (Zhang et al., 2018), the physicochemical properties of rhizosphere soil were measured, including total N, total P, total K, available N, available P, available K, organic matter, and pH. Acid phosphatase activity, invertase activity (Li et al., 2004), catalase activity (Mueller et al., 1997), proteinase activity, and urease activity (Cordero et al., 2019) were measured by the disodium phosphate benzene colorimetric method, sodium thiosulfate titration, permanganate titration, ninhydrin colorimetry, and indophenol blue colorimetry, respectively.

### 2.6 Sample preparation and extraction

#### 2.6.1 Metabolite extraction from roots of peanut and sugarcane

The freeze-dried roots were crushed using a mixer mill (MM 400, Retsch) with zirconia beads for 1.5 min at 30 Hz. Then, samples of powder weighing 100 mg were weighed and extracted overnight at 4°C with 1.2 mL 70% aqueous methanol. Following centrifugation at 12,000 rpm for 10 min, the extracts were filtered. The obtained filtrate was analyzed by UPLC−MS/MS.

#### 2.6.2 Metabolite extraction from rhizosphere soils

Fifty milligrams of rhizosphere soil was homogenized in 500 µL of ice-cold methanol/water (70%, v/v) at 30 Hz for 2 min. After homogenization, the mixture was shaken for 5 min and placed on ice for 15 min. The mixture was centrifuged at 12,000 rpm at 4°C for 10 min, and 400 µL of the supernatant was placed into another centrifuge tube. A total of 500 µL of ethyl acetate/methanol (v/v, 1:3) was added to the original centrifuge tube, and the mixture was oscillated for 5 min and incubated on ice for 15 min. Then, the sample was centrifuged at 12,000 rpm and 4°C for 10 min, and 400 µL of supernatant was transferred into the supernatant above. The two supernatants were pooled and concentrated. Then, 100 µL of 70% methanol water was added to the dried product, and ultrasonic treatment was performed for 3 min. We collected the supernatant by centrifugation at 12,000 rpm for 3 min at 4°C and aspirated 60 µL of supernatant for LC−MS/MS analysis.

### 2.7 Metabolomic analysis

#### 2.7.1 Widely targeted metabolomics

Analytical separation was achieved on an Agilent SB-C18 column (1.8 µm, 2.1 mm*100 mm), and the mobile phases were pure water with 0.1% formic acid (solvent A) and acetonitrile with 0.1% formic acid (solvent B). Separation was performed with a gradient elution program starting conditions of 95% A and 5% B. Within 9 min, a linear gradient to 5% A and 95% B was programmed, and a composition of 5% A and 95% B was maintained for 1 min. Subsequently, a composition of 95% A and 5.0% B was adjusted within 1.10 min and held for 2.9 min. The column oven was set to 40°C, and the injection volume was 2 µL. The effluent was alternatively connected to an ESI-triple quadrupole-linear ion trap (QTRAP)-MS.

LIT and triple quadrupole (QQQ) scans were acquired on a triple quadrupole-linear ion trap mass spectrometer (Q TRAP), AB6500 Q TRAP UPLC/MS/MS system, equipped with an ESI turbo ion-spray interface, operating in positive and negative ionization modes and controlled by Analyst 1.6.3 software (AB Sciex). The ESI source operation parameters were as follows: ion source, turbo spray; source temperature, 550°C; ion spray voltage (IS), 5500 V (positive ion mode)/-4500 V (negative ion mode); ion source gas I (GSI), gas II (GSII), and curtain gas (CUR), 50, 60, and 25.0 psi, respectively; and collision gas (CAD), high. Instrument tuning and mass calibration were performed with 10 and 100 μmol/L polypropylene glycol solutions in QQQ and LIT modes, respectively. QQQ scans were acquired as MRM experiments with collision gas (nitrogen) set to medium. DP and CE for individual MRM transitions were performed with further DP and CE optimization. A specific set of MRM transitions was monitored for each period according to the metabolites eluted within this period.

#### 2.7.2 Nontargeted metabolomics

Untargeted metabolomics profiling was performed using UPLC-Q-TOF/MS (AB SCIEX, MA, USA). The chromatographic separation system was equipped with a Waters ACQUITY UPLC HSS T3 column (1.8 µm, 2.1 mm *100 mm, Waters Co.). The UPLC conditions were as follows: column temperature, 35°C; flow rate, 0.35 mL/min; and injection volume, 1 µL. The mobile phase consisted of 0.04% formic acid in water (phase A) and 0.04% formic acid in acetonitrile (phase B). The linear gradient program was as follows: 95% to 5% phase An over 10 min, holding 5% for 1 min, and 5% to 95% in 0.1 min, holding 95% phase A for 3.9 min.

The mass spectrometer was operated in positive/negative polarity mode with the following settings: Nitrogen was used as the drying gas, nebulizer gas and sheath gas, and the flow was maintained at 8 L/min, while the sheath flow was at 11 L/min. The drying gas and sheath gas temperature was maintained at 325°C. The ESI+ and ESI- voltages were set at 2500 V and 1500 V, respectively. The mass range was set at m/z 50-1700, and the resolution was 30,000 (FWHM). The mass spectrometer was calibrated daily in the mass range m/z 100-1700 before starting the sample analysis by using Agilent tune mix (Part no G1969-85000). The mass accuracy values were good over the entire scan range (mass error < 5 ppm).

### 2.8 Identification and quantification of metabolites

Metabolite profiling was carried out using a widely targeted metabolomics method based on the self-built MWDB database. Substances were qualitatively analyzed according to the secondary spectrum information, and the multiple reaction monitoring mode (MRM) of the triple quadrupole mass spectrometer was used to quantify metabolites.

The original data file obtained by nontargeted metabolomics analysis was first converted into mzML format by ProteoWizard software. Peak extraction, alignment and retention time correction were performed by the XCMS program. The “SVR” method was used to correct the peak area. The peaks were filtered with a deletion rate > 50% in each group of samples. Then, metabolic identification information was obtained by searching the laboratory’s self-built database and integrating the public database and metDNA.

### 2.9 Statistical analysis

Statistical analysis was performed using IBM SPSS Statistics for Windows v. 20 (IMB Corp.) and mean comparisons among treatments were performed using a pairwise comparison test based on S−N-K’s method at a significance level of 0.05. Metabolite data were log_2_-transformed for statistical analysis to improve normality and were normalized. HCA was performed with the R package pheatmap, and the results are presented as heatmaps with dendrograms. Through a principal component analysis, an orthogonal partial least squares discriminant analysis model and differential metabolites were screened with p value < 0.05, FC (fold change) ≥ 1.50 or ≤ 0.67, and VIP (variable importance in project) ≥ 1. Finally, the KEGG database was used for the pathway enrichment analysis of differential metabolites. The correlation and visualization between differential metabolites from rhizosphere soils and soil properties in different treatments were conducted using R software (Version 3.2.1) and visualized using the pheatmap package in R software (Version 3.2.1).

## 3 Results

### 3.1 Soil physicochemical properties under different planting patterns of peanut and sugarcane

Compared with MP, the available N, total P and total K contents, pH, available P, and available K contents of IP increased significantly by 49.1%, 33.8%, 20.3%, 11.9%, 8.0%, and 5.8%, respectively. Compared with MS, the total P content of IS increased significantly by 42.5%, while the available P content decreased significantly by 4.6%. (**Table 1**). The results showed that intercropping treatment can significantly improve soil nutrients.

**Table 1:** Soil physicochemical properties of the tested rhizosphere soils.

Treatment	Available N (mg/kg)	Available P (mg/kg)	Available K (mg/kg)	Total N (g/kg)	Total P (g/kg)	Total K (g/kg)	Organic matter (g/kg)	pH
MP	114.667 ± 3.077b	64.14 ± 0.182b	144.667 ± 5.869b	0.381 ± 0.043ab	0.154 ± 0.004d	3.362 ± 0.257c	34.312 ± 0.63ab	6.413 ± 0.228b
IP	171.00 ± 8.343a	69.274 ± 1.31a	153.00 ± 6.542a	0.272 ± 0.038b	0.206 ± 0.008b	4.046 ± 0.221b	36.613 ± 1.296a	7.173 ± 0.073a
MS	101.444 ± 1.601c	68.665 ± 2.67a	151.556 ± 2.509a	0.381 ± 0.113ab	0.179 ± 0.009c	5.490 ± 0.173a	32.876 ± 4.645b	7.028 ± 0.013a
IS	103.222 ± 5.058c	65.482 ± 2.075b	153.833 ± 2.483a	0.445 ± 0.159ab	0.255 ± 0.014a	5.416 ± 0.249a	31.695 ± 0.474b	7.113 ± 0.019a

MP, MS, IP and IS represent monoculture peanut, monoculture sugarcane, intercropping peanut and intercropping sugarcane, respectively. Six biological replicates were used in this experiment, and values followed by the same letter in the same column are not significantly different (S-N-K’s test, α = 0.05).

**Figure 2:**
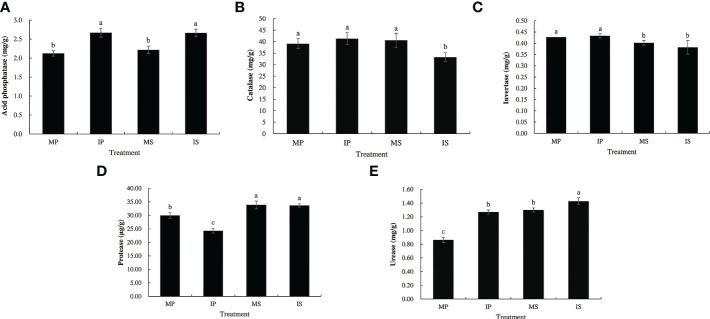
Five enzyme activity of rhizosphere soil in different planting patterns. MP, MS, IP and IS represent monoculture peanut, monoculture sugarcane, intercropping peanut and intercropping sugarcane, respectively. **(A)** Acid phosphatase; **(B)** catalase; **(C)** invertase; **(D)** protease; **(E)** urease. Six biological replicates were used in this experiment, and same letters represents not significantly different (S-N-K’s test, α = 0.05).

There were significant differences in soil enzyme activities between the monoculture and intercropping treatments (**Figure 2**). Compared with MP and MS, IP and IS significantly increased acid phosphatase activity by 25.7% and 20.1%, and urease activity by 47.2% and 9.9%, respectively. However, protease activity decreased significantly by 22.3% in IP, and catalase activity decreased significantly by 18.1% in IS. The soil enzyme assay revealed that the sugarcane/peanut intercropping system contributed to increased acid phosphatase and urease activities but decreased protease and catalase activities.

### 3.2 Metabolite profiles of the different crop roots and their rhizosphere soils

**Figure 3:**
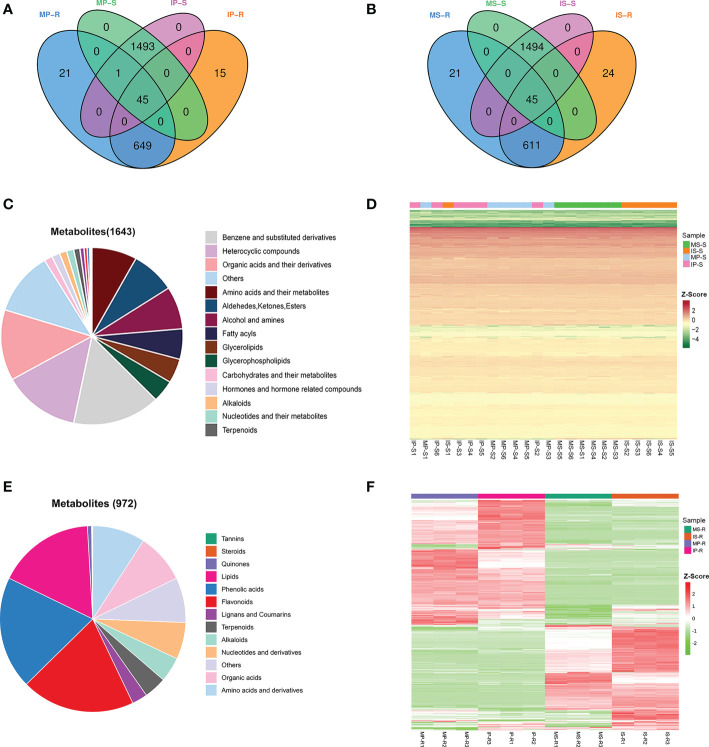
Variability of 972 and 1643 diverse metabolites in the roots and their rhizosphere soils. **(A)** Venn diagram of the root and rhizosphere soil metabolites in monoculture peanut and intercropping peanut; **(B)** venn diagram of root and rhizosphere soil metabolites in monoculture sugarcane and intercropping sugarcane; **(C)** classification of the 1643 identified metabolites in the rhizosphere soils; **(D)** heatmaps of metabolites from rhizosphere soil compared between different groups; **(E)** classification of the 972 identified metabolites in the roots; **(F)** heatmaps of metabolites from roots compared between different groups. Six biological replicates were used to detect the metabolites of MP-S, MS-S, IP-S and IS-S, and three biological replicates were used to detect the metabolites of MP-R, MS-R, IP-R and IS-R.

To detect the variability in metabolites of peanut and sugarcane under monoculture and intercropping systems, we performed metabolome analysis of the roots and their rhizosphere soils. A total of 1643 metabolites were detected from MP-S, MS-S, IP-S and IS-S (**Figures 3A, B**). The 1643 identified metabolites were categorized into 22 categories, among which benzene and substituted derivatives (15.58%), heterocyclic compounds (13.69%), organic acids and their derivatives (12.90%), amino acids and their metabolites (8.03%), aldehydes, and ketones and esters (7.85%) were the dominant metabolites (**Figure 3C**). A total of 972 metabolites were detected from MP-R, MS-R, IP-R, and IS-R and were categorized into 13 categories (**Figures 3A, B, E**). Of these metabolites, the number of flavonoids was the greatest, accounting for 19.75% of the total number of metabolites, followed by lipids (16.87%), phenolic acids (9.16%), amino acids and derivatives (9.16%), and organic acids (8.64%). To clarify the relationship between crop root metabolites and rhizosphere soil metabolites, Venn diagrams of the metabolites were drawn (**Figures 3A, B**). There were 45 metabolites common to MP-S, IP-S, MP-R and IP-R, as well as MS-S, IS-S, MS-R and IS-R. Among them, 32 metabolites were detected in the roots and rhizosphere soils, and organic acids and their derivatives (25.00%) and nucleotides and their metabolites (18.75%) accounted for the largest proportion (**Supplementary Table 1**). The heatmap hierarchical cluster analysis showed that the changes in the contents of the overall metabolite profiles of MP-R, MS-R, IP-R, and IS-R were obviously different, while the contents of the overall metabolite profiles of MP-S, MS-S, IP-S and IS-S were similar (**Figures 3D, F**).

### 3.3 Differential metabolites in the different crop roots and their rhizosphere soils

**Figure 4:**
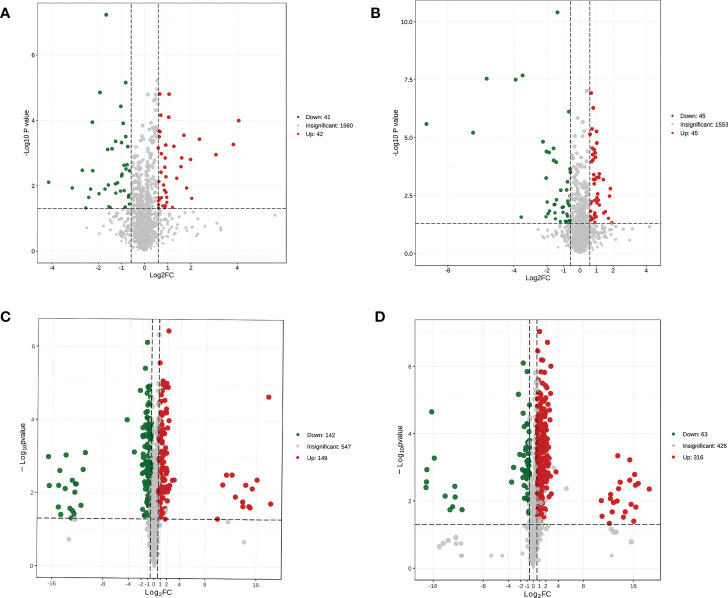
Volcano plot of differential metabolites in the pairwise comparison between. **(A)** MP-S vs. IP-S comparison; **(B)** MS-S vs. IS-S comparison; **(C)** MP-R vs. IP-R comparison; **(D)** MS-R vs. IS-R comparison. Both sides of two dashed vertical lines indicate that the metabolite is down-regulated by less than 1.5 or up-regulated by more than 1.5. Panel **(A, B)** were plotted based on the mean of six replicates, and panel **(C, D)** are plotted based on the mean of three replicates.

To gain insight into the metabolic differences between each group, the results were visualized through volcano plots (**Figure 4**). Eighty-three (42 upregulated) and 90 (45 upregulated) differential metabolites were detected in the MP-S vs. IP-S and MS-S vs. IS-S comparisons, respectively (**Figures 4A, B**). Furthermore, we screened 47 (25 upregulated) and 51 (26 upregulated) significantly differential metabolites with high reliability based on a score > 0.5 (**Supplementary Table 2**). The 47 significantly differential metabolites were glycerolipids (19.15%), organic acids and their derivatives (17.89%), and amino acids and their metabolites (12.77%) accounted for a large proportion in the MP-S vs. IP-S comparison. The 51 significantly differential metabolites heterocyclic compounds (15.69%), glycerolipids (11.76%), organic acids and their derivatives (9.80%), carbohydrates and their metabolites (9.80%), and fatty acyls (9.80%) accounted for a large proportion in the MS-S vs. IS-S comparison. In the MP-R vs. IP-R comparison, 291 (149 upregulated) significantly differential metabolites were detected, among which phenolic acids, flavonoids, and lipids accounted for 20.27%, 17.53% and 16.84%, respectively (**Figure 4C** and **Supplementary Table 2**). In the MS-R vs. IS-R comparison, 379 (316 upregulated) were detected, among which flavonoids, lipids, and phenolic acids accounted for 22.43%, 18.21% and 17.41%, respectively (**Figure 4D** and **Supplementary Table 2**). The results showed that the intercropping system mainly changed the contents of phenolic acids, flavonoids, and lipids in roots, as well as glycerolipids and organic acids and their derivatives in rhizosphere soils. Among the rhizosphere soils, MP-S, MS-S, IP-S and IS-S, the metabolite species were similar, but some metabolite contents were significantly different, such as adenine, adenosine, maltotriose, thermozeaxanthin-13 and PE-NMe (20:0/24:0).

### 3.4 KEGG pathway annotation of the differential metabolites

**Figure 5:**
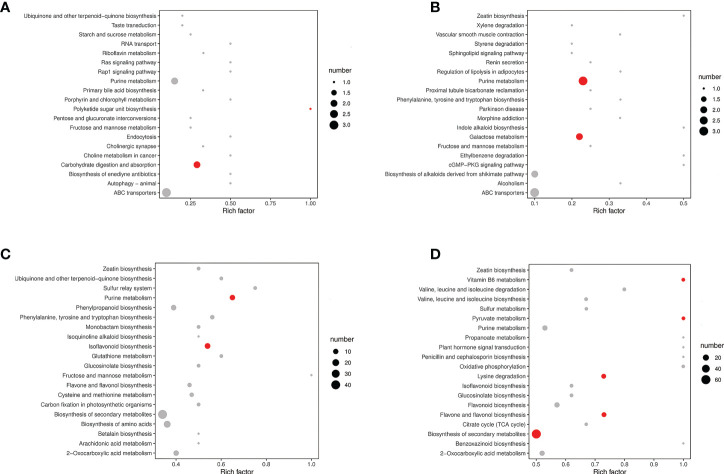
The top 20 pathways of significant of the upregulated and down-regulated differential metabolites on KEGG. **(A)** MP-S vs. IP-S comparison; **(B)** MS-S vs. IS-S comparison; **(C)** MP-R vs. IP-R comparison; **(D)** MS-R vs. IS-R comparison. Red indicated that the pathway was significantly enriched (P < 0.05), and gray indicated that the pathway was not significantly enriched. Panel A and B are plotted based on the mean of six replicates, and panel **(C, D)** were plotted based on the mean of three replicates.

**Figure 6:**
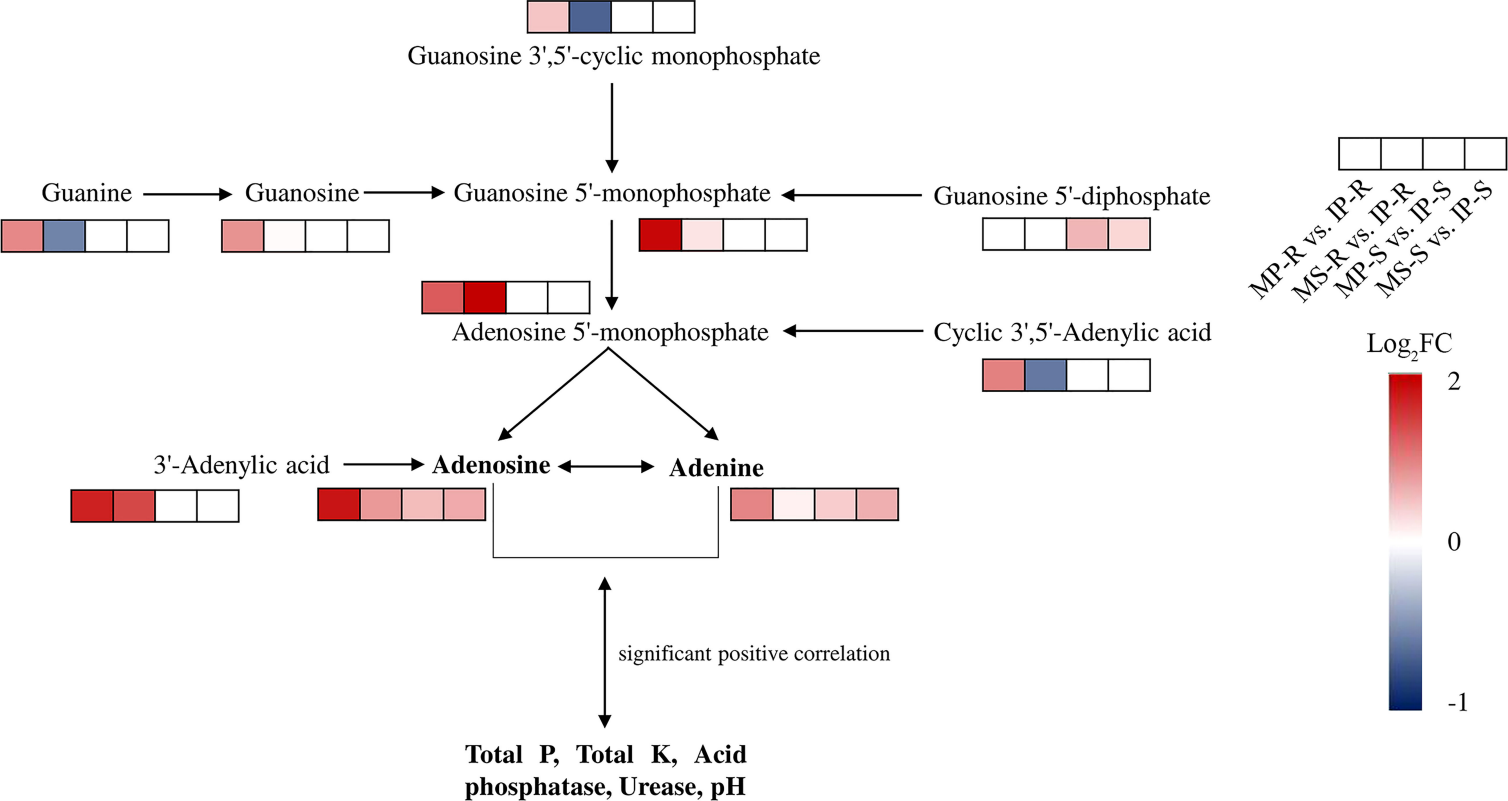
Abundance of the metabolites enriched in purine metabolism compared intercropping treatment with monoculture treatment. The screening of the metabolites was based primarily on one of the comparison groups or multiple comparison groups with a fold change (FC) greater than or less than 1.5, and Log_2_FC processing was performed to draw heatmaps. The FC of MP-R vs. IP-R and MS-R vs. IS-R were mean of three replicates, and the FC of MP-S vs. IP-S and MS-S vs. IS-S were mean of six replicates. The red and blue of small rectangle indicated that up-regulated and down-regulated of metabolite content, respectively; the transparent colorless small rectangle indicated that the metabolite was not detected.

The significantly differential metabolites were annotated using the KEGG database (https://www.kegg.jp/). The top 20 pathways with the highest enrichment in the four comparison groups were identified (**Figure 5**). Carbohydrate digestion and absorption, purine metabolism, and ABC transporters were enriched in the MP-S vs. IP-S comparison (**Figure 5A**). Purine metabolism, ABC transporters, and galactose metabolism were enriched in the MS-S vs. IS-S comparison (**Figure 5B**). Purine metabolism, isoflavonoid biosynthesis, and sulfur relay system were enriched in the MP-R vs. IP-R comparison (**Figure 5C**). Pyruvate metabolism, flavone and flavonol biosynthesis, and biosynthesis of secondary metabolites were enriched in the MS-R vs. IS-R comparison (**Figure 5D**). Among them, 2, 3, 11 and 9 significantly differential metabolites were enriched in purine metabolism in the MP-S vs. IP-S, MS-S vs. IS-S, MP-R vs. IP-R and MS-R vs. IS-R comparisons, respectively. The fold changes in the adenine level were 1.33, 1.55, 1.93, and 1.10, and those in the adenosine level were 1.44, 1.62, 3.50, and 1.75 in the MP-S vs. IP-S, MS-S vs. IS-S, MP-R vs. IP-R and MS-R vs. IS-R comparisons, respectively (**Supplementary Table 1** and **Supplementary Table 2**). That showed adenine and adenosine were detected in the roots and rhizosphere soils, and their levels were increased overall in the intercropping treatment compared to the monoculture treatment (**Figure 6**).

### 3.5 Spearman’s correlations analysis of differential metabolites from rhizosphere soils and soil physicochemical properties

**Figure 7:**
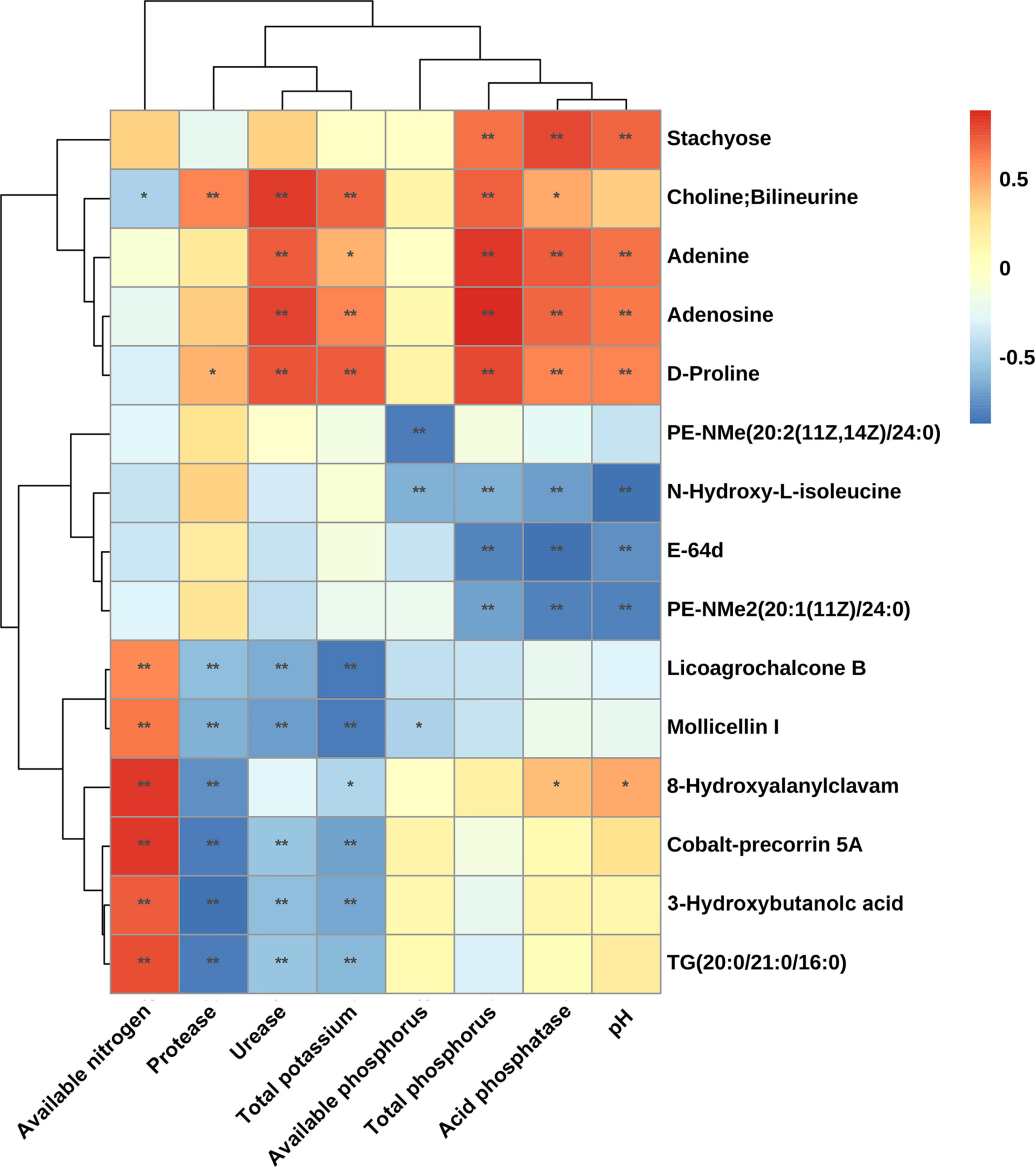
Spearman’s correlations of significant differential metabolites from rhizosphere soils and rhizosphere soil physicochemical properties. Significance between metabolites and physicochemical properties: *, P ≤ 0.05, **, P ≤ 0.01. The figure was plotted based on six repeated values of physicochemical properties of rhizosphere soil and six repeated values of metabolite content.

The correlation between significant differential metabolites in rhizosphere soil and soil physicochemical properties is presented in the form of a matrix heatmap (**Figure 7**). A total of 15 significant differential metabolites were significantly related to eight rhizosphere soil physicochemical properties. Among them, adenine, adenosine, D-proline and choline were significantly positively correlated with total P and total K contents, acid phosphatase and urease activities, and the first three were also significantly positively correlated with pH. N-Hydroxy-L-isoleucine, E-64d, and PE-NMe (20: 2 (11Z, 14Z)/24: 0) levels were significantly negatively correlated with total P content, acid phosphatase activity and pH. Licoagrochalcone B, mollicellin I, cobalt-precorrin 5A, 3-hydroxybutanoic acid and TG (20: 0/21: 0/16: 0) levels were significantly negatively correlated with total K content and urease and protease activities but significantly positively correlated with the available N content. Available phosphorus was only significantly negatively correlated with PE-NMe (20: 2 (11Z, 14Z)/24: 0), N-hydroxy-L-isoleucine and mollicellin I. Both adenine and adenosine were detected in root samples and their rhizosphere soil samples, and their contents were significantly increased in intercropping systems (**Figure 6**). This result indicated that adenine and adenosine in the soil mainly came from root exudation of peanut and sugarcane. The results showed that the accumulation of adenine and adenosine in the soil could be promoted by purine metabolism in the sugarcane/peanut intercropping system, thereby improving the soil physicochemical properties.

## 4 Discussion

Intercropping is thought to be a sustainable agroecological development method that increases land-use efficiency, yields, and soil quality and can also change microclimatic conditions (Agegnehu et al., 2008; Cong et al., 2015). Previous papers have shown that soil nutrients, soil enzyme activities and pH are improved by intercropping systems (Liu et al., 2014; Li et al., 2018). The contents of available N and available K and pH were significantly increased in the cassava/peanut intercropping system (Tang et al., 2020). Pang et al. (2022) and Solanki et al. (2019) found that the sugarcane/peanut intercropping system significantly increased the contents of total P, available P, available K and pH. However, not all of the intercropping systems tended to be better. The content of available N and urease and invertase activities decreased in the tomato/garlic intercropping system. This result indicated that the soil physicochemical properties were affected differently by different intercropped crops (Li et al., 2001). In this study, we found that the contents of total P, total K, available N, available P, available K, and organic matter and pH were significantly increased in intercropping peanut, and the total P content was significantly increased in intercropping sugarcane. Our results were consistent with previous studies showing that the sugarcane/peanut intercropping system had a positive effect on soil nutrition improvement.

Soil enzyme activity is an essential index of soil fertility, quality and health. Soil enzymes interact with the soil environment to indirectly affect soil nutrient levels (Wu et al., 2020). Urease plays an essential role in the nitrogen cycle in soil (Cai et al., 2015), and acid phosphatase activity can be an essential indicator of the hydrolysis of soil phosphorus compounds (Li et al., 2004; Makoi and Ndakidemi, 2008). The results of soil enzyme activity determination showed that the sugarcane/peanut intercropping system significantly increased the activities of urease and acid phosphatase and increased the contents of total P, available N and available P. These results were similar to those of previous studies (Li et al., 2018; Zaeem et al., 2019). In recent years, the sugarcane/peanut intercropping system has become a newly developed cultivation method in South China (Solanki et al., 2019; Tang et al., 2021a; Tang et al., 2021b; Pang et al., 2022). Our results showed that the sugarcane/peanut intercropping system significantly increased the rhizosphere soil nutrients of peanut and had little effect on the rhizosphere soil nutrients of sugarcane.

The root zone is an important microhabitat in soils. Metabolic profiles in root-associated soils are composed of a great variety of chemicals, which recruit specific microbial species to form complex relationships with plants (Kuzyakov and Razavi, 2019). However, little is known about the influence of intercropping on continuous cropping soil metabolites. We found that chemical classes in rhizosphere soil from peanut and sugarcane were highly complex mixtures, including 1643 metabolites, which were categorized into 22 categories. These metabolites were dominated by benzene and substituted derivatives, heterocyclic compounds, and organic acids and their derivatives. In addition, 972 metabolites were identified from the roots of peanut and sugarcane and were classified into 13 categories, including flavonoids, lipids and phenolic acids. The results showed that there were more complex metabolic patterns in rhizosphere soils than in roots, and probably analyzing the rhizosphere microbiome at greater depths will be a potential future research direction. Furthermore, we found that the intercropping system mainly changed the contents of phenolic acids, flavonoids, and lipids in roots, as well as glycerolipids, organic acids and their derivatives in rhizosphere soils. For example, adenine, adenosine, maltotriose, thermozeaxanthin-13 and PE-NMe (20:0/24:0) were significantly increased in the rhizosphere soil of the sugarcane/peanut intercropping system. This observation is in agreement with previous reports on various soils for different plants (Liu et al., 2020; Song et al., 2020). Intercropping is a model of sustainable development of modern agriculture, and using plant allelopathy can provide effective economic and environmental approaches to control successive cropping obstacles (Chen et al., 2017). Compound planting reduced the accumulation of allelochemicals in the soil. Maize-peanut and potato-maize intercropping systems change the type and content of root exudates (Li et al., 2020; Liu et al., 2020).

Most previous studies have shown that soil metabolites can increase nutrient availability in soil (Bais et al., 2006; Holz et al., 2018). However, how complex metabolites in root-associated soils are related to soil physicochemical properties is still largely unclear. This study found that 15 significantly different metabolites from the rhizosphere soils of peanut and sugarcane were significantly associated with eight kinds of rhizosphere soil physicochemical properties. Previous studies have shown that organic acids, including citric acid, malic acid, and oxalic acid, can affect enzymatic activities and increase phosphorus availability in soil (Dakora and Phillips, 2002; Bais et al., 2006). However, our study showed that 42.86% significantly different organic acids in intercropping peanut were upregulated compared with monoculture peanut, and 80% significantly different organic acids in intercropping sugarcane were upregulated compared with monoculture sugarcane. The available P content was significantly increased in IP-S compared with MP-S but significantly decreased in IS-S compared with MS-S. In particular, the oxalic acid content was significantly decreased, while the content of available P from the rhizosphere soil was significantly increased in the MP-S vs. IP-S comparison. In contrast, the oxalic acid content was significantly decreased, while the content of available P was significantly increased in the MS-S vs. IS-S comparison. Previous studies demonstrated that most of the metabolite abundances were higher in rhizosphere soil than in bulk soil (Song et al., 2020). The soil ecosystem is an immensely complex heterogeneous environment governed, and soil microbial community diversity and richness could also affect soil nutrients (Bian et al., 2020; Liu et al., 2020).

The rhizosphere is one of the most dynamic interfaces on Earth, with complex and abundant metabolites (White et al., 2017). These soil metabolites were sourced from plant root exudates, microbial metabolites, and the decomposition of plants, microbes and soil organic matter (Cheng et al., 2018). Currently, differentiating the metabolites from microbe and plant fractions is the greatest challenge for rhizosphere metabolomics (White et al., 2017). Combining nontargeted metabolomics and widely targeted metabolomics, we reported for the first time that 45 common metabolites were detected in the root and rhizosphere soil of peanut, as well as in the root and rhizosphere soil of sugarcane. Among them, 32 are metabolites shared by peanut and sugarcane, with organic acids and their derivatives, nucleotides and their metabolites having the highest proportion. Previous studies have shown that plant root exudates are a highly complex mixture of amino acids, organic acids, sugars, vitamins, purines and nucleosides (Dakora and Phillips, 2002). Hence, we speculate that these metabolites are mainly derived from root exudates from peanuts and sugarcane. In addition, we found that the sugarcane/peanut intercropping system increased the contents of adenosine and adenine in rhizosphere soil by regulating the purine metabolism pathway.

Interestingly, the contents of adenine and adenosine were increased in the sugarcane/peanut intercropping system and were positively correlated with total P, total K, acid phosphatase, urease and pH. The nature of the effect of purine bases on higher plants is unclear, but there have been some reports on the beneficial effects of adenine on root growth. Adenine is important to the biochemistry of every organism because it forms DNA and RNA nucleotides, as well as the energy-rich adenosine triphosphate (ATP) and nicotinamide adenine dinucleotide (NAD). In addition, adenine forms natural CKs (Wróblewska, 2012). However, this compound did not show the inhibitory effect on root development typical for cytokinins (Mathur et al., 2008; Wróblewska, 2012). Adenine can increase root number and rooting percentage when directly applied to the base of cuttings (Wróblewska, 2012). Studies have shown that benzyl adenine, an adenine derivative, has a positive effect on citrus, pea and strawberry. These effective characteristics were manifested in the growth vigor of both roots and shoots, leaf area and yield attributes and significantly increased the contents of nitrogen, phosphorus and potassium in leaves (Al-Juboori, 2016; Aldesuquy et al., 2018; Hussein and Al-Doori, 2021). Similarly, adenosine applied to the root zone of tomato plants significantly increased the total fruit number per plant 1.9- and 2.3-fold and the fruit diameter 1.6- and 1.7-fold (Lovatt et al., 2014). On the other hand, foliar application of adenosine also resulted in a significant increase in yields of clementine mandarin and avocado. Adenosine has been shown to have great potential to increase grower income by increasing fruit size, total yield and fruit quality (Lovatt et al., 2014). Previous studies have shown that adenosine and adenine have a great contribution to promoting plant growth and improving quality and yield, but the effects on soil nutrients have not been reported. Our results showed that the sugarcane/peanut intercropping system promoted purine metabolism, thereby increasing the contents of adenine and adenosine excreted by the roots of peanut and sugarcane into the rhizosphere soils. Furthermore, adenine and adenosine were significantly positively correlated with soil nutrients and enzyme activities. Combined with previous research results, we speculate that adenine and adenosine have the potential to increase soil nutrients and promote crop root development, plant growth, yield increase and quality assurance. However, whether adenine and adenosine have great potential for promoting plant growth by recruiting beneficial microbial communities in the root zones needs to be further explored. This study preliminarily clarified the changes in root and soil metabolites and their relationship with soil physicochemical properties in a sugarcane/peanut intercropping system. The results of this study provide a basis for the mechanistic study of intercropping systems improving soil nutrients.

## 5 Conclusion

Sugarcane/peanut intercropping can improve soil nutrients and enzyme activity. Furthermore, purine metabolism plays a key regulatory role in sugarcane/peanut intercropping by promoting the secretion of adenosine and adenine in roots, accumulating in rhizosphere soil, and improving rhizosphere soil physicochemical properties.

## Data availability statement

The original contributions presented in the study are included in the article/**Supplementary Material**. Further inquiries can be directed to the corresponding authors.

## Author contributions

XT, RT, and SW conceived and designed the experiments and performed the experiments. ZZ, HW, LH, JJ, WM, ZH, FX, JL, RZ, and ZQH assisted in crop cultivation and management and sample collection. XT, RT, SW, and YH analyzed the data, prepared figures and/or tables, and authored or reviewed drafts of the paper. All authors contributed to the article and approved the submitted version.

## Funding

This work was supported by the earmarked fund for National Natural Science Foundation Project (32260544), CARS-13, National Key Research and Development Program (2020YFD1000905), Guangxi Natural Science Foundation Project (2022GXNSFAA035492) and Scientific Project from the Guangxi Academy of Agricultural Sciences (2021YT053&2022YM03).

## Acknowledgments

We thank Dr. Dajie Zhou and Taiyi Yang for helping with the graph drawing.

## Conflict of interest

The authors declare that the research was conducted in the absence of any commercial or financial relationships that could be construed as potential conflict of interest.

## Publisher’s note

All claims expressed in this article are solely those of the authors and do not necessarily represent those of their affiliated organizations, or those of the publisher, the editors and the reviewers. Any product that may be evaluated in this article, or claim that may be made by its manufacturer, is not guaranteed or endorsed by the publisher.

## Supplementary material

Supplementary Table 1 32 metabolites in common in MP-S, IP-S, MP-R and IP-R, MS-S, IS-S, MS-R and IS-R.

Supplementary Table 2 Differentially significant metabolites among different root treatments and different rhizosphere soil treatments.
